# Improving the learning capacity of regional health systems for their transformation towards health and well-being systems: a qualitative study of ten Dutch regions

**DOI:** 10.1108/JHOM-06-2023-0187

**Published:** 2024-08-13

**Authors:** Natascha van Vooren, Esther de Weger, Josefien de Bruin, Caroline Baan

**Affiliations:** Center for Nutrition, Prevention and Health Services, Rijksinstituut voor Volksgezondheid en Milieu, Bilthoven, Netherlands; Tranzo, Tilburg School of Social and Behavioral Sciences, Tilburg University, Tilburg, Netherlands; Athena Institute, Vrije Universiteit Amsterdam, Amsterdam, Netherlands

**Keywords:** Learning capacity, Health system transformation, Cross-sectoral collaboration

## Abstract

**Purpose:**

There is growing recognition that transformation of healthcare systems towards health and well-being systems requires a continuous learning process. This explorative study aims to gain insight into the experiences with and investment in these learning processes within regional partnerships for health and in what they need to enhance their learning capacity to use the learning for transformation.

**Design/methodology/approach:**

17 interviews were held with programme managers, data scientists, trusted advisors and a citizen representative, all involved in the learning process on a regional level in ten Dutch regional partnerships. The interviews were inductively and thematically analysed, focusing on the experiences and perceptions underlying the learning processes.

**Findings:**

Regional partnerships invest in learning processes by organizing interactions between different groups of stakeholders and by reflecting on specific themes or on a region-wide level. Difficulty was found in region-wide reflection and in enhancing the learning capacity within the partnerships. Further enhancing the learning capacity required: (1) Investment in (the use of) expertise for translating learning outcomes into concrete action; (2) Leadership for change, underpinned by a shared sense of urgency to learn for transformation and (3) A facilitative environment for change which is both based on facilitative system structures and a basis of trust and commitment to learn and adapt.

**Originality/value:**

The study highlighted the difficulty of learning on a region-wide level and the struggle to apply this learning for transformation. It provides insights into how learning processes and learning capacity can be further improved.

## Introduction

Western countries worldwide are faced with the difficult and complex task of transforming health systems in order to make them more affordable, accessible and equitable. In pursuit of suitable solutions to build such systems, different types of transformational programmes, such as Accountable Care Organisations (US), Integrated Care Systems (UK), Gesundes Kinzigtal (Germany), the Population Health Management initiatives (the Netherlands) and the Ontario Health Teams (Canada), have been implemented (Alley *et al*., 2016; Ministry of Health, 2022; Pett and Bliss, 2022; Pimperl *et al*., 2017; van Vooren *et al*., 2020). In general, these initiatives comprise local and regional cross-sectoral partnerships for health that aim to reorganize and integrate services across public health, healthcare, social care and wider public services to achieve the Triple Aim (improving experience of care, improving the health of populations and reducing healthcare costs) as part of a larger health systems transformation towards health and well-being systems (Berwick *et al*., 2008; Steenkamer *et al.*, 2020a). For this health systems transformation, there is not a linear trajectory from problem to solution. Instead, health systems transformation requires a continuous process of learning and adapting (Best *et al*., 2012; Holmes *et al*., 2017; Willems, 2021). As such, learning cycles and emergent learning can be seen as pivotal to these local and regional cross-sectoral partnerships (Best *et al*., 2012; de Bruin *et al*., 2022; Steenkamer *et al.*, 2020b; van Vooren *et al*., 2020).

Numerous studies on learning have been performed; however, little concrete connection has been made between learning processes and transformation (Armitage *et al*., 2008; Mierlo and Beers, 2020). The process of learning for transformations requires not only the development of knowledge and interaction with other actors but also the unlearning of old processes (van Mierlo and Beers, 2020). Mierlo and Beers (2020) have compared the value of four learning traditions to gain an in-depth understanding of how learning can be conceptualized in sustainability transitions. Based on these lessons, Mierlo and Beers (2020) suggest two modes of learning for transitions: discursive interaction and reflective action. Discursive interaction includes the creation and/or enhancement of a shared cognitive basis, by sharing knowledge, interests and values. With discursive interaction, information, knowledge and meaning are exchanged during and between meetings (Beers *et al*., 2016; Mierlo and Beers, 2020). Reflective action goes beyond the first mode by including the commitment to “actually do(ing) stuff”. It has a longer timeframe based on an iterative process of the cognitive basis, action and reflection (Mierlo and Beers, 2020). These modes provide preliminary ideas of how to use learning processes for transformation.

In the context of health systems specifically, recent studies have investigated how to improve the processes of learning; these are called learning health systems (LHSs) (Institute of Medicine, 2011). A wide variety of LHS have been implemented; they differ in scale, aims and structure (Menear *et al*., 2019; Enticott *et al*., 2021; de Bruin *et al*., 2022). A recent review identified three different learning processes among these LHSs. According to this review, learning is mostly pursued with knowledge exchange between clinical and research domains or as a cyclical improvement process from data to knowledge, knowledge to performance and performance to data (de Bruin *et al*., 2022). Fewer articles discuss the role of interaction between the different stakeholders in the learning process, and little is known about how partnerships can enhance their collective learning processes in practice (de Bruin *et al*., 2022; Milligan and Berta, 2021). Interaction, however, is found to be a key part of learning for transformation (Beers *et al*., 2016; Mierlo and Beers, 2020).

Despite the emerging field of inquiry in LHSs, not much is known about how learning processes between stakeholders are being applied within regional cross-sectoral partnerships and how these learning processes are translated to action in the context of transformation, which we also refer to as learning capacity (Armitage *et al*., 2008; Medema *et al*., 2014; Lavis *et al.*, 2018; Menear *et al*., 2019). To support regional partnerships to reflect on and enhance the learning capacity in their own regional partnerships, this explorative study therefore aimed to gain more insight into how regional cross-sectoral partnerships for health invest in their learning processes and what is needed to enhance their learning capacity. This study therefore investigated the experiences, implemented strategies and needs of stakeholders from ten Dutch regional partnerships for health regarding their learning processes and learning capacity. This study’s research questions were as follows:

1. What are the experiences with organizing and being part of learning processes within regional partnerships for health?
2. What do stakeholders need to further enhance the learning capacity of regional partnerships for health?

## Conceptual framework

Currently most literature and theory in LHSs conceptualizes learning as either an (intermittent) information exchange between clinical domain and research domain or as a continuous circular process of converting (routine care) data to knowledge, knowledge to performance and performance to data (de Bruin *et al*., 2022). In the context of regional cross-sector partnerships for health, this perspective is too narrowly focused on healthcare instead of health systems, and little attention is given to discursive learning processes among stakeholders (Lavis *et al*., 2018; Menear *et al*., 2019; Lannon *et al*., 2021; de Bruin *et al*., 2022). This study therefore aims for a broadened perspective on learning, including learning among stakeholders of the regional partnerships. This perspective on learning processes is influenced by the integrative approach of Beers *et al*., (2016), which combines both social learning theory and collaborative learning theory (Beers *et al*., 2016). This approach includes the wide, multi-perspective focus from social learning, with the more operationalized processes of how learning in groups takes place (e.g. discursive interaction) from collaborative learning. As such, we define learning processes as the ways of working to connect knowledge, actors and actions to provide learning outcomes. Learning outcomes can be, for example, insight into the progress of the partnership, a broadened or changed perspective or new ideas for change.

In addition, as described before, previously little connection has been made between learning theories and transformation literature (Mierlo and Beers, 2020). Clarity about how the link between learning and transformation is conceptualized in this study is therefore vital. We define the link between learning processes and transformation as “learning capacity”, which is based on the study of Mierlo and Beers (2020).

The term learning capacity refers to the extent to which individuals and organisations within the system are able to (jointly) learn and translate the outcomes of these learning processes to actions of change, thus adapting, to pursue the transformation towards sustainable health and well-being systems (Mierlo and Beers, 2020; Kunseler, *et al*. (2020). Based on literature, we perceive learning capacity to both include processes related to interaction (e.g. shared understanding, shared aims and joint conversations) as well as the use of data infrastructure and evaluation (e.g. shared data infrastructures and intervention monitoring).

Finally, we need to operationalize the meaning of transformation in the context of health systems transformation. Transformation is a “complex, non-linear and radical process of change which leads to a new system” (Minne, van der *et al*., 2021, p. 5). In the context of health systems this is the collaborative process of change that regional partnerships go through to develop from health systems towards health and well-being systems (van Vooren *et al*., 2020). For this study, we focused on transformation on a systemic level, meaning far-reaching changes in regional partnerships’ culture, processes and structures at the different levels (e.g. from citizens and professionals to organisations and communities to cross-organisations and regions) (Embuldeniya *et al*., 2021; Best *et al*., 2012).

## Methods

### Setting

In the Netherlands, there are over 118 regional cross-sector partnerships for health (RIVM, 2021). This study includes ten of these partnerships and is part of a four-year reflexive evaluation named Right care at the Right place (which focuses on the transformation from health systems to health and well-being systems) (RIVM, 2020) in the Netherlands from 2019–2023. As part of this overarching evaluation, ten Dutch regional partnerships were involved in this evaluation to be able to learn with them about their experiences while transforming towards sustainable health and wellbeing systems. These ten regional partnerships were selected based on their variety of scope (rural/urban, regional scope or city scope and varying population sizes of 15,000–800,000 citizens), stakeholders and developmental phase. By selecting this variety of partnerships in our evaluation, we aimed to provide insights related to a broad scope of experiences across regional cross-sector partnerships in the Netherlands.

The involvement of these ten partnerships within the reflexive evaluation provided an opportunity to perform this study, namely to learn from these partnerships regarding their experiences with and strategies for enhancing the learning capacity. Through these interviews, we also aimed to enable participants to reflect on their own learning capacity and how it could be improved to help them develop their health systems.

### Study sample and recruitment

For this study, we performed purposive sampling (Patton, 2002), by which the authors interviewed the programme managers of all ten partnerships that are involved in the reflexive evaluation. The programme managers were expected to have a main role in operationalizing the regional learning processes in the partnerships. We discussed with the programme managers which other relevant actors within their regions to recruit by snowball sampling (Naderifar *et al*., 2017) to get better insights into the experiences with learning capacity. All approached participants agreed to take part in interviews and had given consent. Ultimately, a total of 12 interviews were held with a total of 17 participants – 12 programme managers, two data scientists, one knowledge manager, one researcher and one citizen representative. Interviews were conducted until the authors agreed the point of data saturation had been reached, when no new themes emerged and there was a high rate of recurrence of responses. Some programme managers elected to have dual interviews with other professionals who were involved in creating and supporting the learning capacity, to help enable their mutual reflection on how the learning capacity and processes were run currently and how they would like to improve these *(See* Appendix *for more information on the regional partnerships and the participants).*

### Data collection

To aid data analysis, all interviews were recorded and transcribed verbatim. All study participants had received an information letter and had provided their informed consent. The study received ethics approval from Tilburg University (RP 252), and the interviews were held between May and September 2022. All interviews lasted about one hour, and all but one were performed via video call. For data collection, a semistructured topic guide was used. Participants were asked about their strategies or ways of working, concerning learning processes and what was needed to use these learning processes to aid their transformation process (See Appendix for the interview guide).

### Data analysis

To examine the strategies being implemented within the ten Dutch regional partnerships, the study took a largely inductive approach to thematic analysis. The thematic analysis approach applied within this study followed the six steps outlined in Braun and Clarke (2006). Previous literature and theories on learning processes and strategies that were used to inform the interview questions were also used, in part, to inform the initial coding tree (See Appendix for the translated version of the coding tree). During the coding process, we inductively created a set of final clusters to more fully portray the learning processes and strategies within the Dutch partnerships, thus expanding previous theories. The software programme MaxQDA 2022 was used to code and cluster data following an inductive, sequential and iterative process:

1. NvV and EdW coded relevant sections from interview transcripts and checked each others’ codes (discussing and resolving any discrepancies together);
2. NvV and EdW then thematically analysed, discussed and reviewed the codes in order to create initial clusters.
3. A final draft of the clustered themes was presented to all authors to confirm and refine the themes.

## Results

The following section will describe the results from the interviews in two parts. First, interviewees’ experiences of implementing and enhancing the learning processes within their regional partnerships and their experiences of taking part in the learning process themselves will be described. Based on these experiences, three clusters regarding the investment in learning processes within the regional partnerships were identified: organizing interaction between stakeholders, choosing whom to include in the learning and strategies used for thematic and region-wide reflection. The themes are presented separately (for the readability of the paper); however, it should be noted that these themes are fluid and interact with when it comes to the learning capacity of the regional partnerships. Secondly, we will describe what participants felt was needed to enhance their learning capacity for transformation.

### 1. Experiences with learning processes in the regional partnerships

#### Organizing interaction between stakeholders

Several programme managers highlighted the use of so-called inspiration meetings or knowledge sessions to aid the interaction between professionals, managers and sometimes board members to help them learn about, for example, other perspectives. Some of these meetings were centred on a specific theme or case. Others enabled the exchanging of ideas within a specific group of participants, for example, all project leaders in the partnership. One programme manager explicitly mentioned the difference between inspiration meetings and brainstorm meetings. The latter was not only aimed at understanding different perspectives but also involved the generation of ideas for action. Interviewees felt these meetings were helpful either because they helped the different organisations to understand each other better or because participants got a chance to improve collaboration on specific themes. However, some of them also highlighted several barriers. A common experience was that it remained unclear what and how these learning sessions contributed to the transformation or how to capitalise on the sessions:

It’s a little … You try to fan the flames. But it’s … it’s not immediately obvious; you can’t immediately measure it [the impact/outcome]. You try to get something concrete from these meetings, like: “so what did you learn?” But it’s very difficult to quantify these personal learnings. And you think, you hope, after several of these sessions, there’s the hope that something just starts happening with the different organisations’ (Participant 3).

Reversely, other programme managers felt that the effect of learning by interaction does not always need to be quantified or institutionalized and that the focus should be on creating connections between people to enable the exchange of their ideas and experiences. Regardless of how they thought about the impact or outcomes of such sessions, all interviewees were still searching for how to ensure the learning processes contribute to the transformation of the regional partnerships. They were still searching for ways to ensure that learning led to a change in stakeholders’ and organisations’ behaviours and ways of working, thus enhancing the learning capacity.

In contrast to officially organized meetings, several programme managers had mentioned the importance of learning by doing. Specifically, two programme managers found that they did not have specific strategies for learning on a regional level and stated that they learned by doing based on experiences within their projects instead, and that learning does not always have to be organized.

Trial and error is also a part of it … and I think that the barriers and struggles that you experience during one project, well, you take that with you to the next project. You take that into account when you start a new project. So that you don’t encounter the same barriers. Or when you do, you know how to deal with them (Participant 10)

#### Choosing whom to include in the learning

Most inspiration meetings discussed by the programme managers included a specific target audience. For example, groups of professionals, project leaders or board members. However, programme managers also provided examples of connecting several of these target groups in one session and including additional perspectives, like those of citizens, in the sessions to ensure a more inclusive learning process. Programme managers had different experiences with including multiple perspectives in learning sessions. One programme manager mentioned to have positive experiences with involving different perspectives (professionals, management and citizens) in a theme-focused meeting. However, another programme manager reflected on the complexity of including all those perspectives to come to action. Instead, this programme manager chose to act as a linking pin between the different perspectives to aid the collective knowledge development.

Furthermore, some regional partnerships have mentioned the use and importance of a whole-system-in-the-room approach on a regional level. This means that representatives from the health sector and wellbeing sector, and in fewer cases, citizen representatives, were involved in the planning and decision-making on a regional partnership level.

The idea of the “coalitions” [in the regional partnership] is that you just have the whole-system in the room. So yeah, that all bits of the regional partnership are represented by someone (Participant 4).

The idea behind this approach was that such a broad representation of all actors in the region was needed to sustainably transform the health system for the region and that the variety in perspectives was useful for creating solutions for regional issues. One interviewee discussed the value of including other sectors, like the business sector, in the learning process. This interviewee expected that their different perspectives and ways of working would help improve the learning process.

#### Strategies for thematic or region-wide reflection

Interviewees also talked about their levels of learning. In their regional partnership, most of the learning processes were related to a thematic reflection and optimization process, e.g. improving district nursing. Several programme managers mentioned the use of data to gain insight into project outcomes and processes relating to specific themes. Examples of these were a dashboard which provided insight into projects’ progress, updates within annual reports and periodical evaluation updates with project leaders. Some programme managers mentioned the use of smaller pilots in specific neighbourhoods as another source of reflection. Starting small, e.g. with the use of pilots, was used by several programme managers to learn on a smaller-scale and to build trust and relationships between a selected group of professionals.

Because it’s hard to handle otherwise. All sorts of partners that don’t involve us. Let’s first concern ourselves with our own business, because that’s difficult enough […]I hear the partners also talking about the manageability of it. So that’s why I make it [the learning] smaller, it’s easier that way. However, I’m not sure if that’s easier. Because if the learning becomes more concrete, when you’re really starting to learn and reflect on something, the discussion becomes more difficult at the same time too. (Participant 3).

Another programme manager warned that the pilots, though smaller in scale, are not necessarily easier, as they had experienced that the scaling up of such pilots required more than a copy-paste job. For pilots to be rolled out in other neighbourhoods, they would need to be tailored to the different contextual factors within these neighbourhoods.

Reflection based on outcomes at a regional level was perceived as more difficult due to the experienced limited ability to use and share data on an inter-organizational or regional level. Interviewees also found it difficult to ascribe regional trends to the regional partnership itself.

What you see, for example, you see trends in specific parts of the city where people are getting healthier. But you can’t tell whether that’s because the partnership put a lot of effort into this, or because all the unhealthy people can no longer afford the rent and leave the city. Right, so that remains difficult to tell. So you can measure all sorts of things on a city-wide level, but to ascertain whether those trends are because of all the effort the partnership is putting into it … you just miss things to be able to do that. We just don’t have that yet. So we miss some evaluation and monitoring in that sense and to adjust our projects. You just don’t know correlation-causation …. I find that very difficult that you can’t say with certainty in two years time, whether the partnership has made a difference. (Participant 4).

One programme manager described the experience of a lack of tools, data and expertise to justify the choices made within the regional partnership. As such, some of the regions had started working with trusted third parties. Some regions were working with universities to evaluate interventions implemented on a community-level, while other regions were working together with other data and knowledge institutes to develop and interpret more region-wide-level data.

Interviewees did not just struggle with measuring impact on a regional level; some programme managers also found it difficult to create the sense of urgency for reflection within the regional partnerships. For example, one programme manager argued that the regional partnership was currently more focused on moving forward, e.g. transforming to the next phase of development, and that they therefore experienced little need to learn and reflect together.

We’re still looking ahead instead of reflecting (Participant 5).

Other interviewees had experienced that urgent issues (e.g. COVID-19) often took precedence over the learning process.

But yes, to then have the space and time to sit together with the organisations. Perhaps there’s a role for us [programme managers] to create continuity and a sense of urgency that they must, must, learn together. We’re liable to quickly focus on that one project that needs to be finished, or to be distracted when something new comes by, you know, the urgent issues of the day (Participant 3).

In an attempt to address the lack of urgency for learning, programme managers were looking for a balance between “the learning” and “the doing”. Programme managers discussed the need to find the right frequency of learning within partnerships, e.g. two to four times a year, when new knowledge was available or based on regional partners’ availability and time investment.

### 2. Experienced needs for enhancing the learning capacity

The programme managers had experienced significant difficulties in moving from learning outcomes to actions for transformation, the learning capacity.

I think we’re capable of learning and reflecting with the different organisations. And to find out where the difficulties are. But the next step, like: ‘ok so how are we going to do things now’. That’s, the organisations don’t want to shelve the evaluation, but they’re also not showing different behaviours yet. (Participant 3).

Some programme managers mentioned not knowing how to translate learning outcomes into actions needed for transformation. Also, interviewees discussed the uncertainty of dealing with (financial) system structures, regulations surrounding data sharing and accountability structures that might not fit the changes required for the transformation.

#### A facilitative environment for learning and transformation

To find a way to deal with this uncertainty, programme managers invested in facilitating open and transparent conversations between the collaborative partners. Different from the previously described interactions (e.g. the inspiration meetings), these conversations were focused on the creation of mutual trust and commitment that was needed for the change that organisations would make together. Such strategies were more focused on talking about and committing to shared plans and ideas and sharing each others’ interests and perspectives. Several programme managers have stressed the importance of trust as a basis for openness to change. Though, similar to the previous theme, some programme managers experienced the need to create a balance between “talking and doing” as the organisations within the regional partnership can get bogged down in conversations.

And especially if it’s about the different (conflicting) interests, then you can really get stuck in conversations. That you can’t get past the fact that the one says A and the other B, and then you can get stuck in conversations that can take hours, but ultimately everyone still sticks to their own opinions. That’s what makes it difficult sometimes. Because you don’t progress past those different opinions. (Participant 1).

Furthermore, structures and processes within the current health systems were experienced as hindering the learning processes. Data scientists mentioned that (shared) data infrastructures were, at the time of interviewing, underdeveloped. They found they were unable to share and merge data across different sectors and organisations. This immature data infrastructure, but also the need for skills and expertise to leverage regional-level data, were seen as a barrier for the learning capacity. Additionally, a data scientist felt hampered by national regulations regarding the sharing of data across organizational boundaries.

… that a professionalization needs to happen so that you know what you’re monitoring, why you’re monitoring it, how you’re monitoring it. And for some themes that does happen … But usually there is no real capacity or professional approach to [monitoring]. They try really hard, but there’s a lot of variation (Participant 2)

Some programme managers had experienced the fragmented and short-term funding structures as a barrier to translating learning into action within their regions. A programme manager had mentioned that the short-term funding (geared towards pilots) hampered the scale-up of the learning process based on such pilots. Participants suggested that such experiences highlight the need to invest in regions’ learning capacity by adapting regulations regarding confidentiality and data sharing across organisations and by prioritizing learning and reflecting for transformation (e.g. by hiring additional professionals who have the expertise to develop and leverage shared data infrastructures and who know how to scale up knowledge from pilots).

#### Leadership for change, underpinned by a shared sense of urgency to learn for transformation

Most interviewees had described that a feeling of urgency to change based on reflection is often missing.

When it’s very busy in your own organization, then this collaboration, this urgency is just less present. And yeah, then it’s difficult. Then it’s difficult to even arrange a meeting.

Programme managers were trying to leverage such a sense of urgency in different ways. For example, by actively involving their board members in the preparation for regional meetings or by building mutual trust as a prerequisite for change. Programme managers also invested in regional partners’ awareness of what change would mean for them (e.g. by providing concrete examples of consequences). Leveraging a sense of urgency for change requires a learning process within the individual organisations as well as also the board members of these organisations need to commit to and create room for learning and change. An example was given of whether a municipality is ready to let go of some control and share this with the other regional partners.

If it’s really about creating change, then these people will really need to move past the current organizational boundaries. That requires the right culture and leadership from administrators in those organisations. That’s the key, that’s the next step (Participant 4)

Relatedly, the interviewees were searching for leadership regarding the learning capacity on all levels (both on the board and professional level) to be able to translate the outcomes of learning processes for change.

#### Investing in (the use of) expertise to translate knowledge into concrete actions

Programme managers and data scientists alike mentioned the difficulty of translating learning outcomes into action, as the complexity and scale of the transformation can hold them back from actual change. One programme manager, for example, mentioned the variety of themes that should be taken into account, e.g. finance, data and governance. This complexity and lack of concrete insights into what actions can be taken resulted in a reflex towards more pragmatic actions within the current systems, contrary to actual transformation. Interviewees had experienced that insights about what changes were necessary were difficult to acquire and that translating the insights into action was even more difficult and required expertise (both to properly understand what the insights meant in the local context and to be able to act upon the insights).

Several programme managers reflected on the potential use of data to provide such insights. However, they had experienced difficulties in generating suitable data for decision-making on a regional level. Data scientists highlighted the importance of them collaborating with the programme manager and other regional partners to decide which data is needed for (regional) decision-making. Data scientists had experienced a lack of collaboration with project and programme managers and suspected this was due to all other priorities programme managers and regional partners were facing. Another data scientist had experienced the need to further professionalize the data-infrastructure in the regional partnership including the different organisations. They had experienced that the absence of such overarching data (structures) prevented them from providing region-wide insights for regional decision-making.

## Discussion

This explorative study investigated how regional cross-sectoral partnerships can improve their learning capacity to transform towards sustainable health and well-being systems. The study explored the experiences regarding the learning processes in ten different regional partnerships for health in the Netherlands. These experiences were related to (1) Organizing interaction between stakeholders, (2) Who should be included in the learning process and (3) Strategies used for thematic and region-wide reflection. Region-wide reflection was difficult, as was enhancing the learning capacity within the partnerships. The interviewees identified need to be able to further improve the learning capacity in the regions. These were (1) Creating a facilitative environment for learning and transformation. (2) The need for leadership for change, underpinned by a shared sense of urgency to learn for transformation and (3) Investment in (the use of) expertise in translating learning outcomes into concrete action.

### Theoretical implications

This study is, to our knowledge, one of the first to identify empirical insights about the investment in learning processes and learning capacity for the transformation of regional partnerships for health towards health and wellbeing systems. First, as current conceptualizations of LHSs were often found too narrow for studying learning processes in regional cross-sectoral partnerships for health (Lavis *et al*., 2018; Menear *et al*., 2019; Lannon *et al.*, 2021; de Bruin *et al*., 2022), this study provides a broadened perspective on learning health (and wellbeing) systems. This study, for example, identified the different ways in which discursive interaction interaction was organized, with whom and on what level (thematic or regional).

In addition, in literature, true understanding of how learning relates to transformation is still limited (Armitage *et al*., 2008; Mierlo and Beers, 2020). This study explicitly linked the learning processes to learning capacity as a means to adapt in the context of health systems transformation. This adds an understanding of the variety of contextual factors influencing the use of learning outcomes for transformation. In our study, we found that learning largely happens on specific themes that regions agreed to collaborate on (e.g. mental health financial debt services), but that learning on a regional level to transform the health systems is still very limited. The biggest hurdle seems to be moving from learning to undertaking action based on that learning. This despite the fact that this study shows that acting on the learning is crucial for the desired transformation. Instead, the study and the broader LHS literature indicate that regions focus more on the optimisation of their current ways of working instead – perhaps because it does not require further reaching cultural or structural changes.

### Practical implications

The study also provided the opportunity to reflect on how learning processes and capacities within regional partnerships can be further improved. In doing so, it shows why regions struggle to learn on a systemic level for transformation. Firstly, transformation requires a different mindset underpinned by a willingness and leadership to change, critical self-reflection and vulnerability, as it can mean the destabilization of certain services (Stanlick, 2015). Despite the increasing urgency for transformation in the Netherlands because of rising health and care costs, shortages of healthcare staff and changing healthcare demands (Lorenzoni *et al*., 2019; RIVM, 2018; WHO, 2017, 2021), our results show that the learning in the regional partnerships for health still mainly focuses on the optimization (of current services) instead of learning for real transformation. The relatively low sense of urgency for learning for transformation is not surprising, as there is currently little leadership or accountability for actual transformation of the regional partnerships (RIVM, 2021). Stakeholders are thus able to take part in the learning processes, such as brainstorming sessions, without actually needing to undertake any action or implement any transformational changes. Organizing learning within a regional partnership, therefore, does not naturally mean that this learning is used for transformation. In fact, maintaining a focus on optimization can hamper transformational purposes (Olsson *et al*., 2006). While policy and literature are increasingly ascribing a role for learning in transformation (Mierlo and Beers, 2020; NHS, 2019; Rotmans and Loorbach, 2009; VWS, 2018), an understanding of the required mindset and the willingness, leadership and accountability for the learning capacity to adapt in favour of the transformation should be advocated for.

Secondly, this study also suggests that for regional partnerships to learn and adapt, they require a facilitative environment for their learning processes to enable their transformation. For example, our interviewees stressed that they felt held back by the lack of region-wide data infrastructure and suitable long-term financing to pursue region-wide reflection or build on their pilots. This experience of going back and forth between learning and the capabilities for transformation within the current system is a well-known characteristic of transformation (Darling *et al*., 2016; Rotmans and Loorbach, 2009; Stanlick, 2015). Partnerships need to be reflexive, in other words, be able to react and influence their (systemic) environment (Beers and Mierlo, 2017). This requires a connection between the regional partnerships and the national health system, as the learning endeavours of the partnerships can influence national changes and vice versa. Interestingly, the expressed needs for national changes were all related to systemic changes (e.g. data infrastructure and financing). However, for regional partnerships to be able to learn and change/transform, both in the organization, regionally and nationally, they will also need to create the space to learn, change and make mistakes, especially given the iterative nature of transformation (RIVM, 2021; Rotmans and Loorbach, 2009; Stanlick, 2015).

### Limitations

A limitation of this study might have been the complexity of conceptualizing learning capacity and transformation in practice. This resonates with the experiences in this study, as our interviewees found it difficult to discern and connect the concepts of learning processes and transformation. For example, participants, when asked about their learning strategies, also referred to transformation strategies (e.g. convincing collaborative partners of the importance of systems change). This is perhaps in part because participants did not yet have the required expertise for and knowledge about region-wide learning and therefore found it more difficult to build upon learning as part of their transformation. As such, to improve the learning capacity for the transformation, it is important to invest in regions’ ability and expertise to understand and act on the outcomes of their learning processes. Further research providing examples from practice of how to make a connection between these concepts is needed to understand what strategies are needed to be able to use learning as a means for transformation.

Also, by asking programme managers explicitly about their strategies for learning, we largely focused on the more formal and organized learning strategies instead of examining the time and space for informal learning that may be happening on the workfloor or in communities. Some of our participants rightly pointed out that much learning happens at unofficial “water cooler moments” or simply by gaining more experience on the job.

### Future research

To build on this study’s findings, new research could investigate how to improve the learning capacity by examining and supporting informal learning and how such learning relates to transformation. This would help show how to create space for experiential knowledge (of both professionals and citizens) and to develop reflective action skills. Studies could have a more ethnographic approach, following the development and (in)formal learning activities over time. Future studies could also further examine how to create a facilitating environment to enhance the learning capacity for regional transformation. Such studies could apply a reflexive evaluation study design by which policy, research and practice can cyclically learn and adapt based on first-hand experience of enhancing learning capacity (how to translate learning into action).

## Conclusion

This study described the experiences with learning processes within ten Dutch regional partnerships for health. It also highlighted the difficulty of region-wide reflection and applying learning outcomes for transformation. To enhance this learning capacity, this study provides insights into how learning processes and learning capacity can be further improved. As our study focused on explicitly defined strategies for learning, further research should also examine and support the informal learning activities within regional cross-sectoral partnerships.

## Appendix

Table A1

**Table A1:** Study sample

Region	Goal	Partners	Programmes	Participants in study
Region 1	• Maintaining quality of care and a reduction in waiting lists despite increase in demand • Triple Aim	Municipality, healthcare insurers, various health and care organisations	• Prevention and health care • Improved governance structures • Innovation and improved staff satisfaction	• Programme manager • Data scientist
Region 2	• Happy and health citizens • Transforming from framing healthcare systems from curing disease to caring for neighbourhoods	• Community-led initiatives, municipality, public health organization, knowledge institutes, regulatory body, healthcare insurers, health and care organizations	• Better collaboration between social care and healthcare in neighbourhoods • Prevention • From healthcare to health	• Programme manager • Researcher
Region 3	• 5% more health for citizens in the region within five years (compared to national average) • Finding structural solutions for shifting care from primary care towards community care	• Patient and Public Involvement organizations (PPIs), municipality, health and care organizations, pharmacy and local sport clubs	• Resilient citizens • Positive health for all citizens • Region wide collaboration	• Programme manager • Citizen representative
Region 4	• Vital citizens and vital professionals • Positive health for all citizens • Transforming from framing healthcare systems from curing disease to caring for neighbourhoods and improving citizens’ healthy behaviours	• PPIs, municipalities, public health organization, health and care organizations and project management	• Healthy lifestyle programmes	• Programme manager
Region 5	• Developing a shared vision to ensure all health and care organisations provide the right care and the right place • Working together to ensure patient-centredness • Delivering health and care as close to patients’ homes as possible • Ensuring good quality care remains accessible	• Citizens and patients, municipalities, public health organization, regional ambulance services, health insurer, health and care organisations	• Integrated care pathways • Elderly care • Acute care	• Programme manager
Region 6	• Improving acute care for vulnerable older people • Delivering care closer to citizens’ homes	• Municipalities, health and care organisations	• Coordination of care • Prevention of care • Improving the transfer of care • Improving care at night	• Programme manager
Region 7	• Happy and healthy citizens • Developing shared responsibility for citizens’ health and happiness	• Municipalities, health and care organisations, knowledge institutes and employers	• People centredness • Suitable housing • Healthy and strong cultures	• Two programme managers • Data scientist
Region 8	• Sustainable care for and with citizens • Bringing health rates up to national average • Improving quality of care • Reducing healthcare consumption and costs to national average • Improving staff satisfaction	• PPIs, municipalities, public health care organization, health insurers, health and care organizations	• Regional care and cure • Elderly care • Central triage service • Emergency care • Regional care point	• Programme manager • Knowledge manager
Region 9	• Happy and health citizens • Improving quality and accessibility of care • Reducing (health) disparity rates • Think big and start small	• Municipality, PPI, health and care organizations, project management and health insurer	• Elderly care • Chronic care • Mental healthcare • Youth and family • Digital care • Employment and education • The growing city • Acute care	• Programme manager
Region 10	• Developing a collaborative network to provide the right care at the right place for all citizens	• Municipalities, health insurer, public health organization, financial support services, health and care organizations	• Youth care • Integrated elderly care services • Debt management and poverty • Integrated care for mental health • Prevention and health promotion • Healthcare insurance policy for low income groups	• Programme manager • Data scientist

**Source(s):** Written by the authors NvV and EdW and based on information from the Reflexive Evaluation Right Care at the Right Place, which provided the setting for this study
